# PROTOCOL: The effects of resettlement/re‐entry services on crime and violence in children and youth: A systematic review

**DOI:** 10.1002/cl2.1304

**Published:** 2023-01-10

**Authors:** Jennifer S. Wong, Chelsey Lee, Natalie Beck

**Affiliations:** ^1^ School of Criminology Simon Fraser University Burnaby British Columbia Canada

## Abstract

This is the protocol for a Campbell systematic review. The goal of the study is to examine the impacts of aftercare/resettlement interventions on youth with respect to criminogenic outcomes, and to examine factors related to intervention success. Specific objectives are as follows: (1) What is the impact of aftercare/resettlement interventions on youth with respect to outcomes of crime and violence? (2) How is the treatment effect of aftercare/resettlement interventions on crime and violence outcomes moderated by factors such as participant (e.g., age, race, ethnicity, sex, offender type), treatment (e.g., intensity and quality of implementation), methodological (e.g., measurement of crime, study design, timing of follow‐up measures), and study characteristics (e.g., date of publication, peer‐reviewed status)? (3) Are some types of aftercare/resettlement interventions more effective than others? (4) What are the barriers and facilitators to effective implementation of aftercare/resettlement interventions? (5) What are the mechanisms (theory of change) underlying aftercare/resettlement interventions? (6) What does the available research suggest regarding the cost of aftercare/resettlement interventions?

## 1 BACKGROUND

### 1.1 Description of the problem

Rates of post‐release offending for young custody‐leavers^1^ are high across the global west. For example, the UK House of Commons notes that 69.3% of those under age 18 reoffend within one year of being released from custody (House of Commons Justice Select Committee, 2021). In Australia, longitudinal data show that 41% of youth placed under supervision between 2000 and 2020 received additional sentences before turning 18; for those youth whose first sentence was custodial (versus community‐based), rates were 10% higher (Australian Institute of Health and Welfare, 2021). European studies have found similar rates: in Germany, the reconviction rates of offenders aged 14–15 were estimated to be 46% from 2007 to 2010 (Jehle, 2014). National youth recidivism rates are not available for Canada or the United States; however, provincial and state rates indicate similar levels of recidivism. For example, approximately 83% of incarcerated youth in British Columbia reoffend in the five years following their first offense (British Columbia Ministry of Child and Family Development, 2021), while 76% of Californian youth are rearrested within three years of release (Grassel et al., 2019). Aftercare/re‐entry/resettlement approaches^2^ support individuals as they leave custody and re‐enter their communities (Caputo, 2004). Generally, re‐entry/resettlement programs include supervision as well as any service that is deemed to assist in the successful transition and reintegration of persons from custody to the community (Petersilia, 2004). Examples include assisting young people with access to safe accommodation, facilitating education and job training opportunities, and linking youth with community‐based substance abuse and counseling services (e.g., Barton et al., 2008; Bergseth & McDonald, 2007; Sontheimer & Goodstein, 1993).

Despite earlier summative evidence suggesting positive effects of re‐entry/resettlement services on youth reoffending (Bouchard & Wong, 2018; Weaver & Campbell, 2015), recidivism rates remain unacceptably high, and the positive effects of such programs are often short‐lived (Hazel & Bateman, 2020). Recent reports suggest there may be significant programmatic gaps in meeting the reintegration needs of young custody‐leavers (House of Commons Justice Select Committee, 2021). Such gaps may include, for example, a lack of supports for education, housing, and job training, a failure to focus on internal narrative shifts and pro‐social identities, practices that are not gender‐informed, and a lack of inter‐agency collaboration that support young people upon release (Bateman & Hazel, 2015b). Establishing an updated, comprehensive assessment of the overall impact of re‐entry/resettlement programs on recidivism is paramount to providing precise estimates of the effectiveness of these approaches. Additionally, it is critical to determine how service components, features of program implementation, and participant characteristics are linked with stronger and weaker outcomes.

### 1.2 Description of the intervention

Young people face multiple barriers and challenges post‐custodial release as they return to the community, often making it difficult to reintegrate into their home environments. Although some youth are able to successfully navigate the transition into a stable, prosocial life (Abrams, 2006; McCuish et al., 2018; Todis et al., 2001), many releasees struggle with successful reintegration. Youth sentenced to incarceration are completely removed from their home lives, disrupting and potentially halting any support they may have had from families, friends, and communities that could benefit the process of re‐entry planning (Mears & Travis, 2004; Ruch & Yoder, 2018). This experience is further complicated by the dual transition that young offenders face, as they may be simultaneously transitioning from adolescence to adulthood (Altschuler & Brash, 2004).

Youth are confronted with numerous obstacles throughout the re‐entry process, including barriers related to their physical, psychological, educational, employment, and social needs—many of which are known to increase the risk of recidivism (Barrett et al., 2010; Fields & Abrams, 2010; Kubek et al., 2020; Mears & Travis, 2004). Young offenders have been found to experience higher rates of mental and behavioral health needs in comparison to non‐offender populations (Cauffman, 2004; Fazel et al., 2008). Qualitative research suggests that young people view accessing mental health care as a daunting process, with systemic delays and minimal resources impacting their motivation and ability to seek help and access services (Aalsma et al., 2014). Adolescents also face numerous challenges regarding education and employment opportunities, such as labeling and stigma, a persistent pro‐offending identity, resistance and discrimination, negative attitudes from those in administrative positions, and an overall lack of support (Kubek et al., 2020). Young offenders have also noted social and personal obstacles, such as stress, anxiety, and fatigue (Bateman & Hazel, 2015a), difficulties avoiding the negative and anti‐social peers and activities prevalent in their lives prior to their incarceration, and challenges in cultivating pro‐social friends and connections (Abrams, 2006). In addition, young people may face challenges accessing health care, substance use and addiction support, stable and safe housing, reliable transportation, and resources for developing skills to establish pro‐social and supportive relationships with friends, family, and other influential adults (Aalsma et al., 2015; Abrams, 2006; Barnert et al., 2020; Bateman & Hazel, 2015b; Ruch & Yoder, 2018; Sinclair et al., 2021; Wright et al., 2012).

Upon release, adolescents face a very different environment outside the rigid structure of closed custodial settings, which can prove disorienting and stress‐inducing (Bateman & Hazel, 2015a). Aftercare/resettlement programs differ from traditional models of post‐custodial supervision in two primary ways: (1) youth experience supervision as well as needs‐based supports and services (traditional post‐custodial supervision may or may not include services), and (2) youth typically participate in services while in custody, during the initial transition to the community, and for a supervised period post‐release from custody (Petersilia, 2004; Weaver & Campbell, 2015). While re‐entry programs typically seek to ensure continuity of care throughout the custodial, transition, and post‐release periods, programs vary with respect to the specific components and services offered. In addition to supervision and frequent contacts between case workers and offenders, a common component is intensive case management; this component may include risk and needs assessments to appropriately match the adolescent to relevant supports and ensure that services continue throughout the transition and post‐release supervision periods (Altschuler & Armstrong, 1998). More recently, resettlement policy and literature from the UK has rejected the risk paradigm in favor of approaches that focus on identifying individual strengths and empowering young people to shift to holding prosocial identities (Hazel & Bateman, 2020; Hazel et al., 2017).

Resettlement services for children and youth may include psychological support and treatment, such as cognitive behavioral therapy (e.g., James et al., 2016) or anger management (e.g., Barton et al., 2008), as well as substance use interventions, such as Alcoholics Anonymous or Narcotics Anonymous group sessions (e.g., Bergseth & McDonald, 2007; Bouffard & Bergseth, 2008; Wright et al., 2012). Part of the facilitation process may also include case workers accompanying the youth to their programming (e.g., Bergseth & McDonald, 2007). Other services may include connecting youth to resources regarding housing and accommodation, such as ensuring that they have a safe and stable environment to return to after release, or helping to coordinate foster care placements (e.g., Barton et al., 2008). Employment and education services are also common, including job placements, vocational training, and school supports or required school attendance (e.g., Barton et al., 2008; Bouffard & Bergseth, 2008; Wiebush, 1993). Providing purposeful or constructive activities that help young people look towards their futures and direct their time towards non‐criminal activities is a staple of the resettlement process (Hazel, 2022; Wright et al., 2012). Additionally, programs may aim to increase relational supports for youth offenders, including mentorship (e.g., Bergseth & McDonald, 2007; Bouffard & Bergseth, 2008) and familial supports, such as family conferencing (e.g., Wiebush, 1993) or family group therapy (e.g., Greenwood & Turner, 1993). Some youth may access a wide array of services and supports as part of their resettlement strategy, while others participate in fewer services.

### 1.3 How the intervention might work

**Figure 1:**
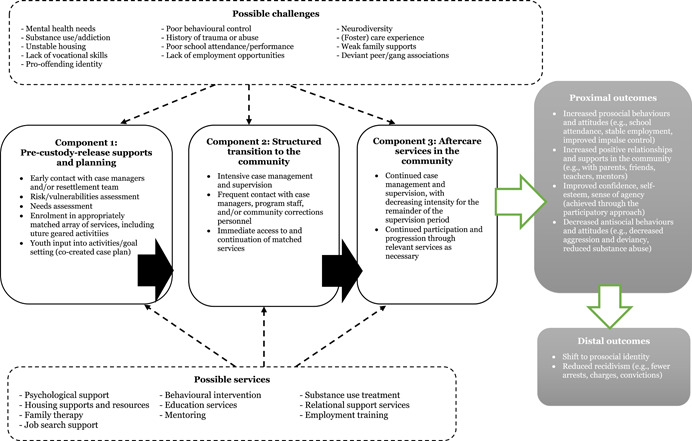
Youth aftercare/resettlement theory of change

Aftercare and resettlement programs were originally developed in response to the need for continued support post‐custodial release and are based on the theoretical underpinning that more supervision and resources will lead to improved outcomes and reduced recidivism. Specifically, programs seek to increase pro‐social identities, opportunities, skillsets, and behaviors (Hazel et al., 2017; Hazel, 2022), while reducing antisocial influences, attitudes, and behaviors (Bouffard & Bergseth, 2008; Chung et al., 2007). Theories of cognitive transformation derived from social control axioms provide some insight into the identity change process that happens during desistance, and stress the importance of individual differences (such as motivation levels and openness to change) and their effects on lasting prosocial behavior (Giordano et al., 2002). In 1988, the U.S. Office of Juvenile Justice and Delinquency Prevention (OJJDP) initiated efforts surrounding juvenile re‐entry concerns, which resulted in the development of the Intensive Aftercare Program (IAP) model (Altschuler & Armstrong, 1994, 2001). Many aftercare programs, including the IAP model, incorporate aspects of strain theories, learning theories, and social control theories (Bergseth, 2010). The IAP model emphasizes three primary components: (1) intervention and rehabilitation programming completed during custody to prepare the individual to reintegrate into the community, (2) a structured transitional process that bridges the institution and community and develops connections between the two environments, and (3) intensive supervision and intervention services provided in the community (Altschuler & Armstrong, 1998). Similarly, UK models stress the importance of five key characteristics during the resettlement process: (1) constructive intervention that encourages the development of a pro‐social identity, (2) co‐created plans that involve the young person, informal support systems, and formal support systems, (3) customized support that takes into account the custody‐leaver's background, strengths, and vulnerabilities, (4) consistent care that spans from (pre)sentencing to successful life in the community, and (5) a coordinated and multi‐agency approach (Bateman & Hazel, 2015b; Hazel et al., 2017; Hazel, 2022). These components are integral to comprehensive resettlement approaches; see Figure 1 for a description of the theory of change.

#### 1.3.1 Custodial phase

The pre‐release stage of aftercare programs provides young people in custody the opportunity to build a strong foundation of skills and behaviors necessary for easing the transition post‐release. Research suggests that successful reintegration is more likely if release planning and intervention programming begin when the individual is admitted into custody, before the start of the transition (Byrnes et al., 2002; Petersilia, 2004; Travis & Petersilia, 2001). In accordance with this principle, the case management component of the aftercare model should ideally commence at entry into custody and continue throughout the supervision period. Important in the case management process is an element of co‐creation; more specifically, by allowing young people to have some self‐determination in their plans for the future, the development of a pro‐social identity may be strengthened (Bateman & Hazel, 2015b). Eligible offenders will generally undergo risk, vulnerabilities, and needs assessments and engage in discussions surrounding their personal goals and motivations, which will inform their case planning with respect to level of supervision and the specific interventions provided while in custody. These interventions will typically focus on preparing the offender to live pro‐socially post‐release, and may include psychological or behavioral interventions (e.g., cognitive behavioral therapy, anger management) or substance use treatment, mental and physical health services, education services, and discharge planning, such as identifying a safe living situation and/or employment preparation and training (e.g., Hampson & Tracey, 2016; James et al., 2013). Further, case planning engages informal networks of support *before* release, in order to provide young people with involved and coordinated support throughout the entire resettlement process (Bateman & Hazel, 2015b).

#### 1.3.2 Transition phase

The immediate period of transition is a critical point in the overall reintegration process. Research suggests that re‐offense is most likely to occur shortly after release, indicating the need for intensive and highly structured transition planning, and immediate access to matched services (Altschuler & Armstrong, 1998; Goodstein & Sontheimer, 1997; Griffin, 2004). As such, aftercare models typically involve intensive case management and supervision immediately after release, with frequent contact between case managers and community correctional staff (Altschuler & Armstrong, 1998; Greenwood & Turner, 1993; Griffin, 2004; James et al., 2016). This can include check‐in meetings, drug testing, family conferencing, and various mentorship activities (Bergseth & McDonald, 2007; Bouffard & Bergseth, 2008; Sontheimer & Goodstein, 1993; Wiebush, 1993). Agencies from all stages of the resettlement process are encouraged to attend meetings during this transitional phase, in order to provide coordinated and seamless care which is unaffected by the spatial changes involved in release (Bateman & Hazel, 2015b; Hampson & Tracey, 2016).

#### 1.3.3 Post‐release phase

After the initial transition to the community has occurred, young custody‐leavers are generally required to maintain contact with their case manager to ensure continued supervision and service participation, including reassessment of needs as necessary (Altschuler & Armstrong, 1998; Griffin, 2004; James et al., 2013). The intensity of this care gradually decreases over time as the young person becomes more established within the community (Griffin, 2004). To successfully implement this phase, it is critical that the resettlement team has established strong connections with community‐based services and resources to ensure that youth receive the appropriate continuity of care to prevent any lapses or delays in service delivery and subsequent skill and behavioral regression (Altschuler & Armstrong, 2001; Mathur & Clark, 2014). The scope of the available services should be broad in an effort to meet the various needs of the adolescents and ensure that services provided in the community parallel those provided in custody (Altschuler & Armstrong, 1998; Byrnes et al., 2002).

### 1.4 Why it is important to do this review

Three prior reviews on the topic of aftercare interventions on recidivism for young offenders have been conducted. First, James et al. (2013) meta‐analyzed 22 studies of experimental and quasi‐experimental reports, with recidivism defined as official reports of re‐arrests and/or reconvictions. The authors found a small, positive overall effect size, suggesting that in comparison to the control group (care as usual), youth who participated in an aftercare program post‐release from a secured facility were significantly less likely to recidivate (*d* = 0.12, *p* < 0.001). Moderator analyses indicated certain characteristics of interventions were related to stronger impacts (i.e., individual treatment, intensive treatment, interventions targeted to high‐risk youth, and interventions targeted to older youth). In addition, well‐implemented programs (as opposed to those noting considerable implementation challenges) were related to stronger outcome effects.

Next, Weaver and Campbell (2015) meta‐analyzed 30 evaluations of youth aftercare programs and found a non‐significant overall treatment impact on recidivism (risk ratio [RR] = 0.931, *p* = 0.117). RRs ranged from 0.391 to 2.095, indicating substantial variability in the effects of the selected interventions. Moderator analyses found that interventions were most effective for youth over the age of 16.5 years, and for youth with a predominantly violent index offense. Similar to James and colleagues, Weaver and Campbell reported that programs without implementation difficulties were related to stronger impacts. In addition, randomized research designs, as well as publication in peer‐reviewed sources, were significant moderators of treatment effectiveness.

Notably, although the reviews by James et al. (2013) and Weaver and Campbell (2015) addressed the same research question, the reviews resulted in conflicting overall conclusions with respect to the impact of aftercare programs on recidivism. This discrepancy is in large part due to differences in author operationalization of “aftercare” interventions, as well as differences in the review inclusion criteria. More specifically, Weaver and Campbell (2015) included studies in which the intervention served specifically as an aftercare approach to shock incarceration and boot camp programs, while James et al. (2013) restricted their set of studies to aftercare programs which “incorporated a treatment modality such as skills training, counseling, and cognitive behavioral therapy” and excluded studies in which the intervention focused on discipline or surveillance (p. 265).

In 2018, Bouchard and Wong published a systematic review and meta‐analysis on the impacts of community‐based aftercare (as well as intensive supervision programs) on criminogenic outcomes for at‐risk youth. The study sought to review the most up‐to‐date research on aftercare programs for youth, and address some of the methodological concerns in the existing literature. For example, the search was restricted to studies published after 1990, which excluded older studies focused on shock incarceration and boot camp programs (which are no longer commonplace). Strict selection criteria were implemented in an effort to increase the generalizability of results to the “typical” offender; further, the authors separated results into “alleged” (e.g., arrests, police contacts) versus “convicted” (e.g., convictions, incarcerations) offenses. In total 10 studies were included, which represented 15 independent program sites. Bouchard and Wong found a positive and significant estimate of treatment impact on alleged offenses (log odds ratios [LORs] = 0.179, *z* = 2.18, *p* = 0.029), but no significant impact for convicted offenses (LOR = −0.029, *z* = 0.27, *p* = 0.784). Moderator analyses indicated that for alleged offenses, weaker research designs, shorter follow‐up periods, and larger sample sizes were related to stronger treatment effects, while for convicted offenses shorter follow‐up periods were also related to stronger impacts. The latter finding suggests that the impact of aftercare/reentry programs may not have a lasting effect when it comes to reducing recidivism.

The current review seeks to update these prior reviews on aftercare programs with the inclusion of new research published in the seven years since Bouchard and Wong conducted their literature search (i.e., in 2015). In addition, the inclusion criteria with respect to intervention type will be expanded to include studies focused on substance abusers, as well as to interventions implemented in both community and closed‐custody settings. Further, the current research will include a qualitative evidence synthesis of process evaluation data from both experimental and non‐experimental studies, a narrative summary of cost‐effectiveness data, and a proposed theory of change (e.g., see White, 2018). The current review will provide decisionmakers with information relevant to considerations of best practices for program components, target population, and approach, and provide evidence for practitioners and policymakers with regards to potential program implementation barriers and facilitators.

## 2 OBJECTIVES

The goal of the study is to examine the impacts of aftercare/resettlement interventions on youth with respect to criminogenic outcomes, and to examine factors related to intervention success. Specific objectives are as follows:

(1) What is the impact of aftercare/resettlement interventions on youth with respect to outcomes of crime and violence?
(2) How is the treatment effect of aftercare/resettlement interventions on crime and violence outcomes moderated by factors such as participant (e.g., age, race, ethnicity, sex, offender type), treatment (e.g., intensity and quality of implementation), methodological (e.g., measurement of crime, study design, timing of follow‐up measures) and study characteristics (e.g., date of publication, peer‐reviewed status)?
(3) Are some types of aftercare/resettlement interventions more effective than others?
(4) What are the barriers and facilitators to effective implementation of aftercare/resettlement interventions?
(5) What are the mechanisms (theory of change) underlying aftercare/resettlement interventions?
(6) What does the available research suggest regarding the cost of aftercare/resettlement interventions?

## 3 METHODS

This review will follow the methodological expectations of Campbell Collaboration intervention reviews (The Methods Group of the Campbell Collaboration, 2019). The review will also be informed by theory‐based impact evaluation (White, 2018), as specified in the theory of change outlined in Figure 1. To address objectives 1, 2, and 3, we will systematically collect and synthesize quantitative outcomes from rigorous impact evaluations of aftercare interventions on young offenders.

To address objective 4, we will collect and examine both quantitative and qualitative research describing the implementation of aftercare interventions, including process evaluations which examine barriers and facilitators to success from the perspectives of both youth and staff. Further, to achieve objective 5 we will expand upon and adapt the theory of change described earlier as necessary based on retrieved research. Finally, to increase the utility of this review for policymakers and other decisionmakers, we will address objective 6 by narratively summarizing available evidence on intervention costs in relation to outcomes and participants served.

### 3.1 Criteria for considering studies for this review

#### 3.1.1 Types of studies

##### Study objectives 1–3

For objectives 1–3, included studies will be restricted to rigorous or moderately rigorous research designs, including randomized controlled trials and quasi‐experimental comparison group designs in which participant baseline variables (e.g., criminal history, age, sex) were examined for observable between‐group differences. Single group pretest‐post‐test designs, as well as comparison group designs deemed weak in baseline matching, will be excluded. Outcomes must include at least one quantitative, individual‐level measure of violence or crime.

##### Study objective 4

For objective 4, included studies will be restricted to implementation assessments of an aftercare intervention, without restriction on study research design, or requirement for quantitative outcomes of violence and crime. Both quantitative and qualitative research will be included. Qualitative studies (or mixed methods studies with a qualitative component) identified through the search will undergo an appraisal of study quality, based on the Critical Appraisal Skills Programme (CASP) qualitative research checklist (2018). Included studies will meet the requirements of questions 1–2 on the checklist; specifically, that the study provides a clear statement of the aims of the research, and that a qualitative methodology was appropriate. See Supporting Information: Appendix B for an overview of the checklist.

##### Study objective 5

Data for objective 5 will be obtained if information on program intervention theory is presented in any of the studies included for objectives 1–4, or if studies relevant to an aftercare/reentry theory of change are identified in the search of gray literature. Included studies will not be restricted to those presenting a program evaluation as long as they present a discussion or description of aftercare/reentry intervention theory of change.

##### Study objective 6

For objective 6, data will be obtained if analyses on program cost‐benefit, cost‐effectiveness, or cost‐per‐participant served are presented in any of the studies included for objectives 1–5. Included studies will not be restricted to those presenting a program evaluation or a discussion of theory of change; studies will be included if they present information on intervention costs in relation to participants served and/or outcomes attained.

#### 3.1.2 Types of participants

Eligible program participants for this review include children and young people who have served or are currently serving a custodial sentence, with an age range of 10–18 years. Studies will also be eligible for inclusion if the participant age range exceeds 18 years (up to 21 years), provided that the average age of study participants is at or below 18. There are no restrictions based on gender, ethnicity, or offense.

Excluded participants include offenders being offered a specialized program based on their exceptional/unique characteristics; for example, those with serious mental health diagnoses (e.g., intellectual disability). Findings from such studies would not be generalizable to broader groups of young people participating in aftercare/resettlement programs.

#### 3.1.3 Types of interventions

Eligible types of interventions include any aftercare/resettlement program that takes place while the young person is in custody, during their transition to the community, and/or when they have returned to the community. The overarching intervention objective should be to promote successful community reintegration and reduce reoffending. Program components may vary across interventions (e.g., intensive case management, housing support, education, counseling), and interventions are not required to contain any particular type of component so long as the intervention is framed as an aftercare/reentry program as opposed to, for example, a more general skills training program or substance abuse recovery program.

Boot camp interventions, wilderness therapy interventions, and intensive supervision programs are excluded from eligibility, as are probation programs that do not meet the definition of resettlement approaches (e.g., those that include only supervision without additional supports and resources). In order to increase the generalizability of findings, programs limited to very specific types of offenders will be excluded, such as specialized programs targeting re‐entry for juvenile sex offenders.

#### 3.1.4 Types of outcomes

##### Primary outcomes

###### Study objectives 1–3

For objectives 1–3, the primary outcomes for the review include individual‐level measures of crime and violent behavior. Outcomes will be categorized into subgroups; these are anticipated to include new arrest, charge, conviction, incarceration (both detention and remand), self‐reported crime, and measures of violence (self‐reported or officially reported). If outcomes are presented in a combined format, they will be coded as a separate subgroup.

Excluded outcomes include status offenses (e.g., truancy, traffic violations) and technical probation violations.

###### Study objective 4

For objective 4, the primary outcomes include any qualitative or quantitative measures related to intervention implementation and process outcomes. Expected outcomes include but are not limited to:

- discussions of gaps in services (e.g., how seamless throughcare services are; whether necessary community‐based services are available),
- participant perceptions of barriers to program engagement,
- practices regarding interagency collaboration and communication (e.g., which data are shared between agencies and how),
- appropriateness/adequacy of staff qualifications and training (e.g., educational requirements, training on risk assessment instruments),
- issues concerning staff or participant safety (e.g., caseworker visits to high‐risk areas),
- resource allocation and casework feasibility (e.g., participant to caseworker ratios; sufficiency of resources for providing a high standard of care),
- program implementation fidelity (e.g., how far the program deviated from its design),
- participant engagement with services and resources (e.g., expected vs. actual service hours or participation; underutilized services; services in high demand), and
- unanticipated challenges with respect to service delivery.

###### Study objective 5

For objective 5, relevant outcomes include any information on intervention theory of change, such as causal pathways between program inputs and expected outcomes.

###### Study objective 6

The primary outcomes for objective 6 include information concerning intervention cost in relation to outcomes achieved, such as a cost‐benefit analysis or cost‐effectiveness analysis, or cost data such as annual program cost in relation to number of clients served.

##### Secondary outcomes

For objectives 1–3, no additional outcomes will be considered.

For objective 4, qualitative data that do not fit within the *a priori* outcomes categories will be coded inductively, allowing other relevant outcomes to present themselves.

No additional outcomes are relevant for objectives 5–6.

#### 3.1.5 Duration of follow‐up

No restrictions will be placed on the duration of participant follow‐up; however, the timing of measurement of quantitative outcomes for objectives 1–3 will be kept as homogenous as possible to maximize between‐study commensurability. For example, if the large majority of studies use a 6‐month follow‐up period, the main outcome time point across all studies will be six months when available (even if some studies include longer periods).

To the extent that some studies include multiple follow‐up points, such as 6‐month, 1‐year, and 2‐year, we will also code outcomes for longer follow‐ups and attempt analyses of smaller pools of studies with different outcome measurement time periods (longer and/or shorter).

#### 3.1.6 Types of settings

No restrictions will be placed on intervention settings; however, depending on the different types of settings uncovered in the search, we may choose to limit analyses to groups of interventions with commensurate settings only. In particular, we anticipate a group of studies based on interventions delivered only in a community setting, and another group based on interventions which begin during custody and continue post‐offender release. Careful consideration will be given to the commensurability of interventions offered in these different categories of settings, and a determination will be made regarding the appropriateness of pooling study findings in analyses for objectives 1–3.

A similar approach will be used for objectives 4 and 6. For example, implementation factors may differ based on location of the intervention; for example, attendance may be a larger barrier in the community than in a custodial setting. As such, qualitative analyses may be limited to summarizing findings from evaluations of interventions from comparable locations. Similarly, intervention costs may differ substantially for interventions delivered in a hybrid prison/community setting than solely in the community, and results will be pooled accordingly.

In addition, as social/political systems and approaches to criminal justice vary substantially across nations, to enhance commensurability and practicality of the findings the study must have been conducted in Canada, the United States, Australia, New Zealand, the U.K., or a Western European country.

#### 3.1.7 Other criteria

Studies will be limited to research published since 1992 (i.e., 30 years; to focus on more contemporary types of aftercare/resettlement approaches), and to research published in English, French, or German (given the evaluation team's existing expertise). If studies in other languages are identified through the search processes, they will be retrieved and documented and a decision concerning the necessity for translation services will be made. As noted previously, studies must have been conducted in Canada, the United States, Australia, New Zealand, the U.K., or a Western European country. No restrictions will be implemented based on publication status or type (i.e., peer‐reviewed; government report versus journal article).

### 3.2 Search methods for identification of studies

#### 3.2.1 Electronic searches

To apply a comprehensive search strategy, the following set of key terms will be used in a Boolean search, reflecting four primary constructs: 1) youth/young person, 2) offending, 3) aftercare/resettlement intervention, and 4) evaluation, theory, or cost:

***Construct 1***: (youth* OR juvenile* OR adolesc* OR teen* OR “young offender*” OR “young people” OR “young person*” OR child* OR “early offender”)

***Construct 2***: (crime* OR criminal* OR devian* OR violen* OR delinquen* OR offend* OR offense* OR offence* OR recidiv* OR reoffen* OR breach* OR “technical violation*” OR arrest* OR convict* OR charge* OR incarcer* OR petition* OR adjudicat* OR caution* OR “compliance during supervision” OR “return to custody”)

***Construct 3***: (diversion* OR divert* OR probat* OR parole OR aftercare OR resettlement OR reentry OR “re‐entry” OR “after custod*” OR supervis* OR “graduated sanction*” OR “intermediate sanction*” OR “early release” OR “pretrial release” OR “supervised release” OR wraparound OR reintegrat* OR throughcare OR “local authority care” OR “security training cent*” OR “care leaver*” OR “detention and training order” OR “youth offending team”)

***Construct 4***: (evaluat* OR effect* OR impact* OR outcome* OR trial* OR treat* OR program* OR randomi* OR experiment* OR assess* OR process OR implement* OR fidelity OR “proof of concept” OR “case study” OR “focus group” OR “pilot study” OR “qualitative” OR “formative evaluation” OR “cost benefit” OR “cost effectiveness” OR “cost analysis” OR “benefit cost” OR “theory of change” OR “program* theory” OR “program model” or “logic model” OR “action theory” OR “causal map”)

The complete search strategy will be applied to a series of 26 electronic databases, including various social sciences databases and those more specifically focused on criminology and criminal justice. Terms will be searched in the “abstract,” “title,” and “subject” search fields. The following databases will be searched; those on the same search platform will be combined:

- *EBSCO*:
  ○ Academic Search Premier
  ○ Criminal Justice Abstracts
  ○ EBSCO Open Dissertations
  ○ Education Source
  ○ ERIC
  ○ Medline
  ○ PsycARTICLES
  ○ PsycBOOKS
  ○ PsycINFO
  ○ Social Sciences Abstracts
  ○ Social Sciences Full Text
- *Elsevier*
  ○ Scopus
- Networked Digital Library of Theses and Dissertations (NDLTD)
- *OVID*
  ○ Cochrane Central Register of Controlled Trials
  ○ Cochrane Database of Systematic Reviews
  ○ Database of Abstracts of Reviews of Effects
- *ProQuest*:
  ○ Applied Social Sciences Index and Abstracts (ASSIA)
  ○ Canadian Research Index
  ○ National Criminal Justice Reference Service (NCJRS)
  ○ ProQuest Dissertations and Theses
  ○ Social Services Abstracts
  ○ Sociological Abstracts
  ○ Sociology Database
- Open Access Theses and Dissertations
- Theses Canada
- Web of Science:
  ○ Science Citation Index Expanded (SCI‐EXPANDED)
  ○ Social Sciences Citation Index (SSCI)
  ○ Arts & Humanities Citation Index (AHCI)
  ○ Conference Proceedings Citation Index—Science (CPCI‐S)
  ○ Conference Proceedings Citation Index—Social Sciences & Humanities (CPCI‐SSH)
  ○ Emerging Sources Citation Index (ESCI)

The complete search string example is provided below for databases in ProQuest.

((abstract(youth* OR juvenile* OR adolesc* OR teen* OR “young offender*” OR “young people” OR “young person*” OR child* OR “early offender”) AND abstract(crime* OR criminal* OR devian* OR violen* OR delinquen* OR offend* OR offense* OR offence* OR recidiv* OR reoffen* OR breach* OR “technical violation*” OR arrest* OR convict* OR charge* OR incarcer* OR petition* OR adjudicat* OR caution* OR “compliance during supervision” OR “return to custody”) AND abstract(diversion* OR divert* OR probat* OR parole OR aftercare OR resettlement OR reentry OR “re‐entry” OR “after custod*” OR supervis* OR “graduated sanction*” OR “intermediate sanction*” OR “early release” OR “pretrial release” OR “supervised release” OR wraparound OR reintegrat* OR throughcare OR “local authority care” OR “security training cent*” OR “care leaver*” OR “detention and training order” OR “youth offending team”) AND abstract(evaluat* OR effect* OR impact* OR outcome* OR trial* OR treat* OR program* OR randomi* OR experiment* OR assess* OR process OR implement* OR fidelity OR “proof of concept” OR “case study” OR “focus group” OR “pilot study” OR “qualitative” OR “formative evaluation” OR “cost benefit” OR “cost effectiveness” OR “cost analysis” OR “benefit cost” OR “theory of change” OR “program* theory” OR “program model” OR “logic model” OR “action theory” OR “causal map”))

OR (title(youth* OR juvenile* OR adolesc* OR teen* OR “young offender*” OR “young people” OR “young person*“ OR child* OR “early offender”) AND title(crime* OR criminal* OR devian* OR violen* OR delinquen* OR offend* OR offense* OR offence* OR recidiv* OR reoffen* OR breach* OR “technical violation*” OR arrest* OR convict* OR charge* OR incarcer* OR petition* OR adjudicat* OR caution* OR “compliance during supervision” OR “return to custody”) AND title(diversion* OR divert* OR probat* OR parole OR aftercare OR resettlement OR reentry OR “re‐entry” OR “after custod*” OR supervis* OR “graduated sanction*” OR “intermediate sanction*” OR “early release” OR “pretrial release” OR “supervised release” OR wraparound OR reintegrat* OR throughcare OR “local authority care” OR “security training cent*” OR “care leaver*” OR “detention and training order” OR “youth offending team”) AND title(evaluat* OR effect* OR impact* OR outcome* OR trial* OR treat* OR program* OR randomi* OR experiment* OR assess* OR process OR implement* OR fidelity OR “proof of concept” OR “case study” OR “focus group” OR “pilot study” OR “qualitative” OR “formative evaluation” OR “cost benefit” OR “cost effectiveness” OR “cost analysis” OR “benefit cost” OR “theory of change” OR “program* theory” OR “program model” OR “logic model” OR “action theory” OR “causal map”))

OR (subject (youth* OR juvenile* OR adolesc* OR teen* OR “young offender*” OR “young people” OR “young person*” OR child* OR “early offender”) AND subject (crime* OR criminal* OR devian* OR violen* OR delinquen* OR offend* OR offense* OR offence* OR recidiv* OR reoffen* OR breach* OR “technical violation*” OR arrest* OR convict* OR charge* OR incarcer* OR petition* OR adjudicat* OR caution* OR “compliance during supervision” OR “return to custody”) AND subject (diversion* OR divert* OR probat* OR parole OR aftercare OR resettlement OR reentry OR “re‐entry” OR “after custod*” OR supervis* OR “graduated sanction*” OR “intermediate sanction*” OR “early release” OR “pretrial release” OR “supervised release” OR wraparound OR reintegrat* OR throughcare OR “local authority care” OR “security training cent*” OR “care leaver*” OR “detention and training order” OR “youth offending team”) AND subject (evaluat* OR effect* OR impact* OR outcome* OR trial* OR treat* OR program* OR randomi* OR experiment* OR assess* OR process OR implement* OR fidelity OR “proof of concept” OR “case study” OR “focus group” OR “pilot study” OR “qualitative” OR “formative evaluation” OR “cost benefit” OR “cost effectiveness” OR “cost analysis” OR “benefit cost” OR “theory of change” OR “program* theory” OR “program model” OR “logic model” OR “action theory” OR “causal map”))

Additional web sources will also be searched to identify relevant unpublished works not indexed in academic databases (e.g., technical reports, conference papers, and independent research projects) in an effort to minimize the risk of publication bias. Specifically, we will identify relevant conference and meeting abstracts via the meeting archives of the American Society of Criminology, the British Society of Criminology, the European Society of Criminology, and the Conference Proceedings Citation Index. In addition, the websites of several relevant organizations will be searched, including the Australian Institute of Criminology, Confederation of European Probation, Department of Justice Canada, European Crime Prevention Network, Home Office UK, Ministry of Justice UK, National Institute of Justice, New South Wales Bureau of Crime Statistics and Research, Office of Juvenile Justice and Delinquency Prevention, US Department of Justice, and the Youth Justice Board. Last, a number of open access search engines will be searched, including the Directory of Open Access Journals (DOAJ), Bielefeld Academic Search Engine (BASE), and CrimRxiv.

Search terms on websites, meeting archives, and open access engines will include a reduced set of the terms from the database search strategy and will be adjusted across different sites and search restrictions accordingly. If the site allows for advanced search strategies, we will use multiple combined terms; if the site allows for only basic search strategies, we will attempt a series of searches such as (“young person” and resettlement) or (youth and aftercare).

#### 3.2.2 Searching other resources

Additional search methods will include hand‐searching the reference lists of existing review literature in the field (e.g., Weaver & Campbell, 2015), and the reference lists of all studies meeting inclusion criteria. We will also review the curricula vitae of known researchers in the field (e.g., Tim Bateman; Jeffrey Bouffard; Anne‐Marie Day; Barry Goldson; Kate Gooch; Pippa Goodfellow; Paul gray; Neal Hazel; John Pitts), and we will contact the first authors of included reports to inquire if they are aware of any additional studies. Further, websites specifically affiliated with an aftercare program will be searched for evaluations. The search will also include the tables of contents of the following 25 journals, backdated to 24 months prior to the date of implementation of the electronic database search:

- *British Journal of Criminology*
- *Canadian Journal of Criminology*
- *Canadian Journal of Criminology and Corrections*
- *Canadian Journal of Criminology and Criminal Justice*
- *Corrections: Policy, Practice & Research*
- *Crime and Delinquency*
- *Crime Prevention and Community Safety*
- *Criminal Justice and Behavior*
- *Criminal Justice Review*
- *Criminology & Public Policy*
- *Criminology and Criminal Justice*
- *European Journal of Crime, Criminal Law, and Criminal Justice*
- *European Journal of Criminology*
- *European Journal on Criminal Policy and Research*
- *Federal Probation*
- *International Journal of Offender Therapy and Comparative Criminology*
- *Journal of Community Corrections*
- *Journal of Experimental Criminology*
- *Journal of Offender Rehabilitation*
- *Journal of Research Crime and Delinquency*
- *Juvenile and Family Court Journal*
- *Probation Journal*
- *Residential Treatment for Children & Youth*
- *Youth Justice*
- *Youth Violence and Juvenile Justice*

### 3.3 Data collection and analysis

#### 3.3.1 Description of methods used in primary research

##### Study objectives 1–3

The search is designed to capture all relevant program evaluations of aftercare/resettlement interventions for custodial or post‐custodial youth that meet the aforementioned inclusion/exclusion criteria in the section “criteria for selecting studies for this review” (above). We expect the primary research used to assess intervention effectiveness will utilize randomized controlled designs or quasi‐experimental comparison group designs in which participants were matched on at least some baseline variable, will include at least one quantitative individual‐measure of crime or violence, and will have been conducted in a westernized country, and published after 1991 in English, French, or German (e.g., Barton et al., 2008; Greenwood & Turner, 1993; Hawkins et al., 2009).

##### Study objective 4

Items 1 and 2 on the CASP (2018) qualitative studies checklist will be used to filter studies relevant to objective 4 (see Supporting Information: Appendix B). Specifically, included qualitative studies or mixed method studies which present process evaluations of aftercare/reentry programs will be those that (1) clearly state research goals, and (2) use qualitative methods in a way that can answer the stated research question (CASP, 2018). Only studies conducted in a westernized country, and published after 1991 in English, French, or German will be considered (e.g., Barton et al., 2008; Bergseth & McDonald, 2007).

##### Study objectives 5 and 6

No specific research methods are required for studies included under objective 5 or objective 6, and a variety of quantitative and qualitative study designs are expected (e.g., Bergseth & McDonald, 2007; Greenwood & Turner, 1993). Only studies conducted in a westernized country, and published after 1991 in English, French, or German will be considered.

#### 3.3.2 Selection of studies

The initial database and gray literature search will be split into two sections, with Reviewer 1 conducting the search for the first section, and Reviewer 2 conducting the search for the second section. Specifically, each reviewer will read through the titles and abstracts of identified hits in their designated set of databases/sources to determine studies that appear potentially relevant to the review objectives and to be retrieved in full for further assessment. At this point in the selection process, we will only remove hits that are obviously irrelevant (e.g., book reviews, non‐human studies). The lists from both reviewers will then be combined into a single database, and copies of all studies will be retrieved. Using the combined list, two reviewers will independently apply the inclusion and exclusion criteria to every article in the list to determine whether it meets the full set of selection criteria for objectives 1–3 and/or objectives 4–6. Discrepancies between reviewers will be resolved by a third reviewer.

#### 3.3.3 Data extraction and management

The included studies will be split in half, with one coder conducting primary data extraction for each study in their set. Data will be entered into a Microsoft Excel database. A third coder will independently validate all coding for all studies by cross‐checking the database coding with each research report. Discrepancies between coders will be discussed until consensus is reached.

For objectives 1–3, an extensive coding scheme involving approximately 75 variables will be used to extract data from each study. The coded variables will fall into categories of general study characteristics (e.g., publication type, program delivery year), program components (e.g., vocational skills, family involvement), information about the study sample (e.g., age, racial mix, gender mix, sample size), study characteristics (e.g., research design, baseline group differences, outcome measure, follow‐up period), and outcomes (e.g., means and standard deviations, frequencies, group sample sizes, *F*‐statistics).

For objective 4, additional variables will be used to code qualitative research. Extracted information will relate to study quality (e.g., reporting practices, ethical considerations, limitations), data characteristics (e.g., types of data presented), and process evaluation outcomes (e.g., data on program fidelity, resourcing, or staff qualification).

For objective 5, any information regarding data from intervention cost analyses (e.g., cost‐benefit, cost‐effectiveness, cost in relation to participants served) will be extracted from each study.

For objective 6, any information concerning the intervention's theory of change will be extracted, for example, theorized pathways for participants as they move between program activities towards desired outcomes.

See the draft coding form in Supporting Information: Appendix A for more details.

#### 3.3.4 Assessment of risk of bias

##### Study objectives 1–3

Studies will be assessed by two independent reviewers for potential risk of bias using the Cochrane Risk of Bias in Non‐randomized Studies of Interventions (ROBINS‐I; Sterne et al., 2016) or the Cochrane Risk of Bias tool for randomized studies (RoB‐2; Higgins et al., 2019). Studies will be scored across a series of domains to determine a rating of high, medium, or low risk of bias.

Medium and high risk of bias studies will be carefully reviewed by the team and a decision about retention versus elimination from the analytic sample will be made. See also the section on Sensitivity Analysis for planned testing concerning publication bias and influential studies, as well as the section on Moderator Analyses in which plans are outlined for exploring several study‐related characteristics as potential moderators of treatment impact.

##### Study objective 4

We will assess the quality of included qualitative studies using an adapted version of the CASP qualitative study checklist (CASP, 2018), making judgments on the adequacy of reporting, data collection, presentation, analysis and validity of the conclusions drawn. The checklist is included in Supporting Information: Appendix B. We will exclude studies of particularly low quality at this stage (Noyes et al., 2019), as well as studies in which questions 1–2 are assessed as “No.”

The remaining studies will be classified as of high or low quality and the results of the quality appraisal will be reported in the review (Noyes et al., 2019). Further, as described in the section on Sensitivity Analysis, a sensitivity analysis of qualitative evidence will be performed to assess the contributions of individual studies to the synthesized process evaluation findings.

##### Study objective 5

Assessing the risk of bias is less relevant to studies presenting information on intervention theory of change; however, any study presenting discrepant or conflicting information will be discussed by the research team until a decision about inclusion is reached.

##### Study objective 6

To assess methodological quality and risk of bias, studies will be assessed using the Consensus on Health Economic Criteria (CHEC‐list) developed by Evers et al. (2005). Studies will be rated on a series of 19 criteria to calculate a global assessment of study quality. One point will be allocated for each “yes” rating and 0.5 points for each “suboptimal” rating; total scores per article will be divided by the number of applicable checklist items for the particular study (Wijnen et al., 2017). Studies scoring below 65% will be carefully reviewed by the team and a decision about retention versus elimination from the analytic sample will be made.

#### 3.3.5 Measures of treatment effect

Data from each individual study will be standardized so that results across studies can be meaningfully pooled. Effect size calculation will depend on the type of data presented in each individual study. More specifically, we anticipate the following calculations, although the final list will depend on the results of the study coding^3^:

(a) For studies that present means and standard deviations for both groups at post‐test, the basic standardized mean difference will be calculated as the mean of the treatment group (*M*_*T*_) minus the mean of the control group (*M*_*C*_) divided by the pooled standard deviation (SD_pooled_):
$$\mathrm{Cohen}\prime s\;d=\;\frac{M_{T}-\;M_{C}}{{\mathrm{SD}}_{\mathrm{pooled}}}.$$
  Where the pooled standard deviation will be calculated by:
$${\mathrm{SD}}_{\mathrm{pooled}}=\;\sqrt{\frac{{\left(n_{T}-1\right)}_{T}^{2}+\;{\left(n_{C}-1\right)}_{C}^{2}}{\left(n_{T}-1\right)+\left(n_{C}-1\right)}}.$$
  In which ${\mathrm{SD}}_{T}^{2}$ is the standard deviation of the treatment group and ${\mathrm{SD}}_{C}^{2}$ is the standard deviation of the control group.
  The standard error will be calculated by:
$${\mathrm{SE}}_{d}=\sqrt{\frac{n_{T}+n_{C}}{n_{T}n_{C}}+\frac{d^{2}}{2\left(n_{T}+n_{C}\right)}}\;$$
  To correct for possible small sample bias, we will apply Hedges' correction, resulting in Hedges' *g*:
$$\mathrm{Hedges}\prime g=\;\left[1-\frac{3}{4n-9}\right]\left[\frac{M_{T}-\;M_{C}}{{}_{\mathrm{pooled}}}\right],$$
  where *n* = *n*_*T*_ + *n*_*c*_, *n*_*T*_ and *n*_*T*_ are sample sizes for the treatment and control groups, respectively, and SD_pooled_ is the pooled estimate of the standard deviation of the treatment and control groups.
(b) If the report involves a pretest‐post‐test control group design, we will adapt the formula above to factor in the baseline data obtained at pretest and increase precision, as follows:
$${\mathrm{ES}}_{\mathrm{SMD}}=\left[1-\frac{3}{4\left(n_{T}+n_{C}-2\right)}\right]\left[\frac{\left(M_{T,\mathrm{post}}-\;M_{T,\mathrm{pre}}\right)\left(M_{C,\mathrm{post}}-\;M_{C,\mathrm{pre}}\right)}{{\mathrm{SD}}_{\mathrm{pre}}}\right].$$
  Using a small samples bias corrector, *M*_*t*,post_ is the post‐test mean of the treatment group, *M*_*t,*pre_ is the pretest mean for the treatment group, *M*_*c,*post_ is the post‐test mean for the control group, *M*_*c, pre*_ is the pretest mean for the control group, and SD_pre_ is the pooled standard deviation at pretest (Morris, 2008), calculated as follows:
$${\mathrm{SD}}_{\mathrm{pre}}=\;\sqrt{\frac{{\left(n_{T}-1\right)}_{T,\mathrm{pre}}^{2}+\;{\left(n_{C}-1\right)}_{C,\mathrm{pre}}^{2}}{n_{T}+n_{C}-2}}.$$
  The standard error will be calculated as:
$${\mathrm{SE}}_{\mathrm{SMD}}=\sqrt{\frac{n_{T}+n_{C}}{n_{T}n_{C}}2(1-\rho )+\frac{{\left({\mathrm{ES}}_{\mathrm{SMD}}\right)}^{2}}{2\left(n_{T}+n_{C}\right)}},$$
  where ρ is the population correlation between the pretest and post‐test measures, operationalized as the sample correlation between the baseline and outcome measures if provided in the study report. If the correlation is not available, we will follow the What Works Clearinghouse (2020) and assume a value of 0.5 for ρ.
(c) If a study presents an unstandardized beta coefficient, standard deviation of the dependent variable, and sample sizes, the effect size will be calculated as follows, where SD_pooled_ is calculated using pre‐test *n's*:
$${\mathrm{ES}}_{\beta }=\;\left[1-\frac{3}{4n-9}\right]\left[\frac{\beta }{{\mathrm{SD}}_{\mathrm{pooled}}}\right].$$
  The standard error will calculated by:
$${\mathrm{SE}}_{\beta }=\sqrt{\frac{n_{T}+n_{C}}{n_{T}n_{C}}+\frac{{\left(\mathrm{ES}\right)}^{2}}{2\left(n_{T}+n_{C}\right)}}.$$
(d) If a study presents an *F‐*test with unequal sample group sizes, the effect size will be calculated as follows:
$${\mathrm{ES}}_{F}=\;\sqrt{\frac{F\left(n_{T}+\;n_{C}\right)}{n_{T}n_{C}}},$$
  where *F* is the *F*‐statistic, *n*_*T*_ is the post‐test sample size for the treatment group and *n*_*C*_ is the post‐test sample size for the control group. The standard error will be calculated as per the formula in bullet (c).
(e) For studies that present dichotomous outcome measures (e.g., in the form of percentages or raw numbers representing how many participants were rearrested at least once), effect sizes will be computed as odds ratios. The odds will refer to the odds of recidivism (e.g., arrest, conviction, or incarceration) compared to no recidivism for an individual who participated in an aftercare intervention, relative to the odds of recidivism for an individual in the control group (Lipsey & Wilson, 2001). Specifically:

$$\mathrm{OR}=\;\frac{\mathrm{ad}}{\mathrm{bc}}=\;\frac{P_{a}\;P_{d}}{P_{b}\;P_{c}}=\;\frac{P_{a}\;÷\;P_{b}}{P_{c}\;÷\;P_{d}}=\;\frac{P_{a}(1-p_{c})}{P_{c}(1-p_{a})},$$

where a, b, c and d correspond to the raw frequency of those who recidivated and those who did not for each group. For example, *a* refers to the number of youth in the treatment group who *were* arrested, *d* refers to the number of youth in the treatment group who were not arrested, *b* refers to the number of youth in the comparison group who *were* arrested and *c* refers to the number of youth in the comparison group who were not arrested. The superscript *P* refers to the proportion in the relative cell (*a, b, c, d*) and lower‐case *p* refers to the proportion of persons in its relative group (*a* or *c*) that experienced a positive outcome (reduction in recidivism) (Lipsey & Wilson, 2001).

For ease of interpretation, all odds ratios will be log transformed to LORs; LORs are centered around a value of zero, with zero indicating that recidivism is equally likely to occur in both groups. Data will be coded (or reverse‐coded) such that an LOR below 0 indicates that the outcomes favor the control group (with the treatment group being more likely to recidivate), and a value above 0 indicates that the aftercare intervention has a beneficial impact on the treatment group (a lower rate of recidivism).

Given that LORs (based on dichotomous data) are not numerically compatible with standardized mean differences (based on continuous data), we will adjust by using a Cox transformation (Sanchez‐Meca et al., 2003):

$${\mathrm{ES}}_{\mathrm{cox}}=\;\frac{L(\mathrm{OR})}{1.65}.$$

Where the standard error will be calculated as:

$${\mathrm{SE}}_{\mathrm{cox}}=\sqrt{.367\left[\left(\frac{1}{a}\right)+\left(\frac{1}{c}\right)+\left(\frac{1}{b}\right)+\left(\frac{1}{d}\right)\right]}.$$

If the studies include both pretest and post‐test data, this formula will be adapted to take this information into account. The pretest‐adjusted formula is as follows:

$${\mathrm{ES}}_{\mathrm{cox}}=\;\left[\frac{L_{\mathrm{OR},\mathrm{post}}}{1.65}\right]-\;\left[\frac{L_{\mathrm{OR},\mathrm{pre}}}{1.65}\right].$$

With the standard error calculated as:

$${\mathrm{SE}}_{\mathrm{cox}}=\sqrt{.367\left[\left(\frac{1}{a_{\mathrm{pre}}}\right)+\left(\frac{1}{c_{\mathrm{pre}}}\right)+\left(\frac{1}{b_{\mathrm{pre}}}\right)+\left(\frac{1}{d_{\mathrm{pre}}}\right)\right]}.$$

#### 3.3.6 Unit of analysis issues

##### Study objectives 1–3

If any clustered research designs are uncovered in the analytic sample of studies for objectives 1–3, effect sizes and standard errors will be adjusted accordingly using an estimate of the intra‐cluster correlation coefficient, a measure of the proportional variance attributable to group differences. See Hedges (2006, 2007).

##### Study objectives 4, 5 and 6

Unit of analysis issues with respect to clustered research designs are not relevant for study objectives 4, 5, and 6.

#### 3.3.7 Criteria for determination of independent findings

##### Study objectives 1–3

A key assumption in meta‐analysis is the independence of observations (Card, 2011). With respect to the inclusion of effect sizes for objectives 1–3, to ensure independence several decision rules will be implemented. We note, however, that all relevant effect size data will be extracted from each study, and all potential effect sizes will be calculated:

(a) If multiple reports present data from the same population or study (e.g., a dissertation and a journal article, or two separate articles using overlapping research samples), all available information will be used but the study will only be counted a single time.
(b) If a single report includes multiple experiments, they will be counted as separate studies only if the samples are completely independent and the control group is not double counted.
(c) If a study includes multiple treatment groups (e.g., versions of the aftercare intervention) compared to a single control group, the treatment group that is the most comparable to those in the overall set of studies will be selected for inclusion.
(d) When multiple post‐tests are reported (e.g., an immediate post‐test and a 6‐month follow‐up), the most common time point across all included studies in the set will be chosen.^4^
(e) If studies report multiple outcomes that are categorized under the same outcome measure category (e.g., different measures of violence), the most commensurate outcome to other studies will be selected.

Depending on the nature of the final set of effect sizes, we will consider the use of models using robust variance estimation so that more effect sizes can be included (Pustejovsky & Tipton, 2021; Tanner‐Smith & Tipton, 2014).

##### Study objectives 4 and 6

For study objectives 4 and 6, independence is less of a concern. However, if multiple reports present process evaluation or cost‐benefit data from the same population or study (e.g., a dissertation and a journal article, or two separate articles use overlapping research samples), the one with the most detailed all relevant information will be coded by the study will only be counted one time.

##### Study objective 5

For objective 5, the concept of study independence is not relevant; any information uncovered that can be used to refine the theory of change will be incorporated.

#### 3.3.8 Dealing with missing data

We anticipate substantial “missing data” to be encountered throughout the coding process; specifically, across the set of included studies certain pieces of information may not be presented by study authors (e.g., sample size, participant age, participant gender). We will attempt to obtain this information by contacting study authors; otherwise, when possible, we will calculate the missing data using data available in the evaluation report. For example, a study may report only the total sample size, rather than specifying the sample sizes per treatment/control group. In these cases, when other information regarding the sample size is available such as degrees of freedom from a reported *F‐*test, we will calculate the treatment and control group sample sizes using an assumption of proportional group attrition. If missing quantitative data needed for inclusion in meta‐analysis cannot obtained from study authors and cannot be calculated, the study will be excluded from meta‐analysis and coded as “data not available.”

#### 3.3.9 Data synthesis

##### Study objectives 1–3

The two main approaches to modeling data in meta‐analysis are fixed effects and random effects models. Both models weight each study by its inverse variance, however, the calculation of weights is based on the assumed source of variability between studies (Card 2011; Egger & Smith 2001). A fixed effects model assumes that between‐subject variability present is the result of sampling error and occurs only by chance, while random effects models assume that between‐study heterogeneity is important and is due to factors other than random subject‐level sampling error (Card 2011; Lipsey & Wilson 2001). In the current analysis, given the expectation of a multitude of between‐study differences, we plan to implement DerSimonian and Laird random effects models. All analyses will be conducted in Stata. Results will be presented in forest plots.

##### Study objective 4

For outcome 4, both qualitative and quantitative data will be collected to identify barriers and facilitators to program implementation and fidelity to intervention design. The extraction of qualitative data will be in part based on an *a priori* set of expected findings, derived from a preliminary review of the literature. Specifically, variables, such as custodial intervention programming, planning and preparation services, immediate transition services, and community components will guide coding in a deductive fashion. For reported data that do not fit into these pre‐defined variables, an inductive thematic approach will be taken to code and analyze these findings, allowing new themes and categories to emerge.

Coding will follow the three stages of aftercare: (1) pre‐release programming and planning, including risk assessments and case management, (2) intensive transition structures and processes, including throughcare services, and (3) post‐release supervision and services, including intensive supervision and reintegration programming. See Table 1 for an example of a coding scheme, guided by deductive and inductive themes in each of the three stages of aftercare. A complete list of *a priori* variables can be found in the coding form (Supporting Information: Appendix A).

**Table 1:** Sample coding framework

	Framework categories		
	Pre‐release programming and planning	Intensive transition structures and processes	Post‐release supervision and reintegration
Examples of *a priori* variables	*Example 1*: Staff preparedness	*Example 1*: Continuity of care	*Example 1*: Implementation fidelity
Examples of emergent codes	*Example 1*: Problems with risk assessment practices	*Example 1*: Gaps in communication between custodial and community staff	*Example 1*: Limited treatment referral options
	*Example 2*: Time constraints due to short sentences and preparation programming	*Example 2*: Staff retention and turnover	*Example 2*: Participant lack of consistent attendance at therapy

#### 3.3.10 Assessment of heterogeneity

##### Subgroup analysis and investigation of heterogeneity

###### Study objectives 1–3

Three approaches will be used to assess sources of heterogeneity for objectives 1–3. First, we will examine Cochran's *Q*‐statistics and *I* ^2^ statistics (Higgins et al., 2003). Second, through an examination of the outcome categories used across studies (e.g., rearrest, reconviction) we will disaggregate our pooled outcome measures into smaller, more commensurate sets. As per Bouchard and Wong (2022), meta‐analyses of correctional intervention effectiveness that use aggregated measures of crime/recidivism (i.e., multiple measures combined) versus disaggregated measures (e.g., arrest, conviction, incarceration) may provide an incomplete and potentially misleading assessment of treatment effectiveness.

Third, we will investigate between‐study heterogeneity of effects through subgroup (moderator) analysis to examine whether certain categorical variables can explain some of the variability in effect sizes (Card, 2011; Lipsey & Wilson, 2001). Potential dichotomized variables include: publication year (e.g., prior to vs. post‐2010), publication type (peer reviewed vs. not peer‐reviewed), research design (strong vs. moderate), time of post‐test (immediately after program end vs. 6+ months after program end), sample race (group was predominantly Caucasian/mixed race vs. predominantly racial minority); and inclusion of various program components (e.g., education component (yes vs. no), individual therapy component (yes vs. no), etc.). With respect to race and ethnicity, depending on the data available in individual studies, analyses may be possible for racial and ethnic subgroups as opposed to the aforementioned dichotomization.

Subgroup analysis will be conducted using the analog to the ANOVA method, which separates the total variability (*Q_T_*) into the within‐group variation (*Q_w_* = the summed *Q*‐statistics for each of the two groups in the analysis) and that which can be explained by the categorical variable (the between‐group variation; *Q_b_* = the difference between the total and within *Q*‐statistics). If the *Q_b_* is statistically significant, it suggests the two categories are producing significantly different effect sizes and the difference is due to more than sampling error (Lipsey & Wilson 2001). Associations among moderators will be reported in an effort to address potential collinearity.

In addition, random effects meta‐regression models will be implemented, incorporating multiple moderators in the same model (Hedges & Olkin, 1985; Higgins & Green, 2006). This approach will reduce potential confounding across moderators, and allow for the inclusion of continuous predictors (if coding permits). As in standard linear regression analysis, the coefficients derived from a meta‐regression describe how the dependent variable changes with a unit increase in the predictor (moderator) variables.

###### Study objective 4

Subgroup analysis of qualitative studies will be conducted based on themes uncovered from the process evaluation evidence synthesis for each of the three stages of aftercare. Specifically, overarching themes and findings will be presented, as well as subgroup analysis based on findings related to the custodial phase of intervention, the transition phase, and the post‐custodial phase.

###### Study objective 5

Subgroup analysis and investigation of heterogeneity is not relevant for objective 5 (theory of change).

###### Study objective 6

Depending on the nature of the available data, intervention cost‐related data may be analyzed in smaller sets based on intervention setting (e.g., for those programs delivered in a hybrid prison/community setting than solely in the community), program provider (public vs. private organization), geographic location (United States vs. United Kingdom), and so forth.

#### 3.3.11 Assessment of reporting biases

##### Study objectives 1–3

For objectives 1–3, publication bias (Sterne & Harbord, 2004) and small study effects will be assessed using Egger's test of small study effects and funnel plots (Egger et al., 2003; Steichen 1998; Sterne et al., 2000).

##### Study objectives 4–6

Reporting biases are not considered relevant for study objectives 4, 5, and 6.

#### 3.3.12 Sensitivity analysis

##### Study objectives 1–3

For objectives 1–3, we will test sensitivity of the findings to strongly influential studies by conducting a remove‐one‐study influence analysis (Tobias, 1999). To do so, each study in the meta‐analysis will be omitted, one at a time, and the pooled effect will be recalculated without that study to determine whether its removal has a notable impact on the pooled findings of the meta‐analysis. Further, we will test the sensitivity of findings by removing all studies coded as being at high risk of bias, and recalculating the pooled effect to assess for any notable changes to findings.

##### Study objective 4

A sensitivity analysis of the synthesized process evaluation findings will be performed to assess the contributions of qualitative studies. By examining both the “frequency” (e.g., if certain conclusions are reliant on data from a single study) and “thickness” (e.g., the depth of understanding that studies bring to conclusions) of study outcomes within the qualitative evidence synthesis findings, we will gain insight into the influence and profile of individual studies (i.e., if studies with low or high methodological quality are more influential), and possible biases that shape our synthesis findings (Carroll et al., 2012; Carroll et al., 2013).

For example, in synthesizing qualitative findings regarding interagency communication, we may conclude that case management data are/are not shared consistently between stakeholders within aftercare/reentry programming. However, in assessing the coded data which contribute to this finding, we may find that one study provided most of the evidence regarding interagency communication, and that the removal of this study would alter our conclusion.

In addition, depending on the nature of the findings we will conduct a sensitivity analysis using the CASP (2018) qualitative study quality appraisal checklist as a guide (see Supporting Information: Appendix B). For example, the level of quality observed among qualitative studies (high vs low in methodological quality, based on a cut off score on the quality appraisal checklist) will be used to examine contributions to synthesized process evaluation findings. Where conclusions are based primarily on studies with low methodological quality, we will temper our conclusions appropriately.

###### Study objective 5

Sensitivity analysis is not relevant for objective 5 (theory of change).

###### Study objective 6

Sensitivity analysis is not relevant for objective 6 (intervention cost).

#### 3.3.13 Treatment of qualitative research

The study will synthesize qualitative data by drawing on thematic analysis and framework synthesis (Carroll et al., 2011; Dixon‐Woods, 2011; Thomas & Harden, 2008). Following a systematic search of the literature (using electronic databases and gray literature sources), retrieved qualitative studies will be read and re‐read by two independent reviewers, and findings will be coded into themes based on the initial framework of the aftercare model. Further, as new themes emerge that are not decided *a priori*, the framework will be updated to represent the emerging synthesis. As in previous studies utilizing framework synthesis (e.g., Abbott et al., 2019), our goal is to present an understanding of the effects of aftercare and resettlement programming as well as an explanation of the mechanisms that work to produce these effects. Triangulation between results and findings of the quantitative and qualitative evidence reviews, as well as existing literature, will form the basis of a convergent mixed‐methods review (Noyes et al., 2019, p. 21).

### 3.4 Roles and responsibilities

The team is well suited to carry out the review. Jennifer Wong (JSW) has content expertise on aftercare/reentry programs and has previously published a meta‐analysis on aftercare programs for at‐risk youth (Bouchard & Wong, 2018). In addition, JSW, Chelsey Lee (CL), and Natalie Beck (NB) are all experienced with methods of systematic review and meta‐analysis and possess methodological expertise, statistical expertise, and information retrieval expertise. More specifically, JSW has published 18 manuscripts (and three government reports) which utilize the techniques of systematic review and meta‐analysis (e.g., Bouchard & Wong, 2022; Wong et al., 2019a, 2019b). CL has published five papers focused on systematic review and meta‐analysis (e.g., Lee & Wong, 2022; Wong et al., 2021); while NB recently conducted and published a systematic review and meta‐analysis on the effects of wilderness therapy programs on delinquent youth (Beck & Wong, 2022).

To summarize, the team expertise is as follows:

- Content: JSW
- Systematic review methods: JSW, CL, and NB
- Statistical analysis: JSW, CL, and NB
- Information retrieval: JSW, CL, and NB

The protocol was developed by Jennifer S. Wong (JSW) and Chelsey Lee (CL) with contributions from Natalie Beck (NB). CL and NB will conduct the systematic literature search. Decisions on inclusion for impact evaluation studies will be made by all three team members, with conflicts resolved through discussion until consensus is reached. Qualitative study coding will be carried out by CL and NB and validated by JSW. The estimation of effect sizes will be done by CL and NB and validated by JSW. Reporting writing will be led by JSW and conducted by all three team members.

## SOURCES OF SUPPORT

**Internal sources**

None.

**External sources**

We would like to thank the Cambpell Collaboration and the Youth Endowment Fund for financial support for this review.

## DECLARATIONS OF INTEREST

The lead author has been involved in a prior systematic review on this topic. Otherwise, there are no known conflicts of interest for the research team. Forms attached.

## PRELIMINARY TIMEFRAME

Following is a preliminary timetable with target dates for accomplishing the key tasks required to complete the review; however, our ability to start on tasks may be impacted by the date the protocol is approved.

- Searches for eligible studies: December 23, 2022
- Screening/compiling results from the literature search: January 9, 2023
- Extraction of data from eligible research reports: February 15, 2023
- Statistical analysis: March 15, 2023
- Preparation of the final review report: April 19, 2023

## PLANS FOR UPDATING THE REVIEW

The rate of publication of rigorous studies in this field is likely to be slow. Jennifer Wong will monitor the literature in the field every 5 years and will coordinate an update of the review once sufficient high‐quality studies become available.

## Supporting information

Supporting information.

## Footnotes

1 We note that while North American literature typically uses the terms “youth” and “adolescent,” UK literature more frequently uses the terms “children” and “young person” to refer to individuals in the same general age category.

2 In the UK, a young person's “transition from custody to the community is commonly known as “resettlement,” with practitioner involvement known as resettlement support” (Hazel, 2022). In North American literature, this process is typically referred to as “aftercare” or “re‐entry,” and is often framed as a program, practice, or intervention. In an effort to be inclusive, herein we use all terms synonymously.

3 For example, if a study outcome is presented as a conditional mean estimated by regression, we will use the formulas presented in Taylor et al. (2022) to estimate the effect size.

4 As noted above, outcomes for all follow‐up time points will be coded and, if there are sufficient numbers of outcomes at these additional time points (e.g., 2‐year follow‐up), subgroup meta‐analyses will be conducted.
