# Supplementary material for: PROTOCOL: The effects of resettlement/re‐entry services on crime and violence in children and youth: A systematic review
Source: Campbell Syst Rev. 2023 Jan 10;19(1):e1304. doi: 10.1002/cl2.1304 (PMC9831282; doi:10.1002/cl2.1304)
Supplement: Supplementary file 1 — Supporting information. [file CL2-19-e1304-s001.docx]

APPENDICES

Following is a draft coding form for the review; to be revised as necessary during the coding process.

**Appendix A. Draft Coding Form**

| General study characteristics | |
| --- | --- |
| *Variable* | *Coding details* |
| Author (date) | String variable |
| Outcome number | No specification (e.g., 1, 2, 3, etc.) used when one study contributed more than one outcome effect) |
| Targeted population | 0 = Referred/selected for program  1 = Open to all youth |
| Study publication year | No specification (e.g., 1995, 2001, etc.) |
| Publication type | 0 = Journal article  1 = Book chapter  2 = Report  3 = Dissertation/thesis |
| Peer review | 0 = No  1 = Yes |
| Year(s) of study | No specification (e.g., 2002-2006, 2015, etc.) |
| Location | 0 = North America  1 = UK  2 = Western Europe  3 = Australia/New Zealand |
| Source of funding | String variable |
| Program characteristics | |
| *Variable* | *Coding details* |
| Program name | String variable |
| Program delivery year | No specification (e.g., 1995, 2001, etc.) |
| General program description | String variable |
| Program theory of change | String variable; any information concerning proposed pathways to desired outcomes for participants |
| Program contained behavioral/emotional skill component? | 0 = No  1 = Yes |
| Program contained academic component? | 0 = No  1 = Yes |
| Program contained vocational component? | 0 = No  1 = Yes |
| Program contained life skills training component? | 0 = No  1 = Yes |
| Program contained housing component? | 0 = No  1 = Yes |
| Program contained individual therapy component? | 0 = No  1 = Yes |
| Program contained group therapy component? | 0 = No  1 = Yes |
| Program contained family therapy component? | 0 = No  1 = Yes |
| Program contained mentoring component? | 0 = No  1 = Yes |
| Program contained case management component? | 0 = No  1 = Yes |
| Any other program components not listed | String variable |
| Program duration | No specification (e.g., 8 weeks) |
| Parent/family involvement | 0 = No  1 = Yes |
| Setting | 0 = Community only  1 = Custody only  2 = Custody and community component |
| Delivered by | 0 = Probation officers  1 = Case manager  2 = Police  3 = Counsellor  4 = Program staff  5 = Students  6 = Volunteers  7 = Various community service providers  8 = Other |
| Risk assessment tool used? | 0 = No  1 = Yes |
| Participation mandatory? | 0 = No  1 = Yes |
| Type of offender/risk level | 0 = Low  1 = Medium-High |
| Cost data | No specification; any information related to intervention cost in relation to participants and/or outcomes |
| Notes re. cost data and any potential for bias | String variable re. any potential bias re data/methods used to estimate intervention cost, participants served, intervention benefits, or anything else |
| CHEC-list score | Score on the Consensus on Health Economic Criteria (CHEC-list) and number of applicable checklist items |
| % on the CHEC-list | Score/# of applicable items |
| Additional notes re. program characteristics | String variable |
| Qualitative study characteristics | |
| *Variable* | *Coding details* |
| Type of data presented | String variable (e.g., themes, quotes, narrative) |
| Data collection | String variable (e.g., interview, survey, or observational data) |
| Data coding approach | String variable (e.g., inductive, deductive, axial coding, focused coding) |
| Data analysis | String variable (e.g., thematic analysis, narrative analysis) |
| Credibility issues? | String variable (e.g., sample not appropriately described, coding and analysis procedures not shared) |
| Researcher reflexivity | String variable (e.g., researcher position or biases addressed/identified) |
| Ethical considerations noted | String variable |
| Limitations noted | String variable |
| Process evaluation findings | |
| *Variable* | *Coding details* |
| Continuity of care | String variable (e.g., gaps in services noted) |
| Interagency communication | String variable (e.g., data sharing protocols, single or regular meetings/sessions held involving staff from both custody and community) |
| Staff preparedness | String variable (e.g., inadequate training noted; clear roles/duties/responsibilities laid out) |
| Safety concerns | String variable (e.g., caseworker visits to high-risk areas) |
| Participant perceptions | String variable (e.g., barriers to participation) |
| Resources/funding | String variable (e.g., caseworker to participant ratios, staff turnover and retention) |
| Implementation fidelity | String variable (e.g., underutilized services) |
| Unanticipated challenges | String variable (e.g., with respect to service delivery) |
| Any additional qualitative findings | String variables (to be coded inductively) |
| Score on the CASP | 0 = low quality  1 = high quality |
| Impact evaluation study characteristics | |
| *Variable* | *Coding details* |
| Research design | 0 = RCT  1 = QE w/matched comparison group  2 = QE w/weakly matched comparison group |
| Random assignment? | 0 = No  1 = Yes |
| Control group type | 0 = RCT control  1 = Waitlist control  2 = Matched control |
| Control group description | String variable (e.g., youth returning from custody without any reentry services) |
| Pretest? | 0 = No  1 = Yes |
| Quality of implementation: Any problems noted | 0 = No problems  1 = Minor problems  2 = Major problems |
| Researcher involvement | 0 = Evaluation only  1 = Involved in delivering intervention  2 = Involved in developing intervention  3 = Developed and delivered intervention |
| Declarations of interest among primary researchers | 0 = No  1 = Yes |
| Notes re. research design and any potential for bias | String variable describing any potential concerns with the research design that may lead to biased findings. |
| Score on the ROBINS-I or ROB-2 | 0 = low risk  1 = medium risk  2 = high risk |
| Sample characteristics | |
| *Variable* | *Coding details* |
| N treatment group | No specification (e.g., n=64) |
| N comparison group | No specification (e.g., n=23) |
| Participant age | No specification (e.g., 14-21 years) |
| Approximate mean age | No specification (e.g., 18.2 years) |
| SD of mean age | No specification (e.g., 0.9 years) |
| Gender mix | No specification (e.g., 74% male) |
| Racial mix | No specification (e.g., 17% Black, 24% Hispanic) |
| Ethnicity mix | No specification (e.g., 82% Chinese) |
| Unit of assignment | 0 = Individual  1 = Group |
| Outcomes adjusted for pre-test differences? | 0 = No  1 = Yes |
| Direction and magnitude of initial differences between treatment and control group | String variable (e.g., treatment group significantly higher in proportion male than control group) |
| Attrition from treatment and control groups on recidivism outcome | String variable (e.g., 26% from pretest to post-test) |
| Notes re. study sample and any potential for bias | String variable describing any potential concerns with the study sample that may lead to biased findings (e.g., major attrition in the control group.) |
| Outcome measure | |
| *Variable* | *Coding details* |
| Outcome measure name | String variable (e.g., self-reported crime, Y-OQ, police reports) |
| Direction of measure | 0 = Increase in score is good  1 = Increase in score is bad |
| Source of measure | 0 = Official report  1 = Self-report  2 = Other report (parent, case manager, etc.) |
| Measurement | 0 = Dichotomous  1 = Continuous |
| Time of post test | String variable (e.g., on last day of program) |
| Time of follow-up(s) | String variable (e.g., 6 months following post-test) |
| Findings | String variable (e.g., significant reduction in social problems from pre-test to post-test, no significant differences from post-test to 6-month follow up) |
| Notes re. outcome measures and any potential for bias | String variable describing any potential concerns with the study outcome measures that may lead to biased findings (e.g., change in measurement approach from pretest to post-test). |
| Impact evaluation findings | |
| *Variable* | *Coding details* |
| Treatment group mean/SD at pretest | No specification (e.g., 4.7 (1.2)) |
| Treatment group % at pretest | No specification (e.g., 21%) |
| Treatment group N at pretest | No specification (e.g., 144) |
| Treatment group mean/SD at post-test | No specification (e.g., 4.7 (1.2)) |
| Treatment group % at post-test | No specification (e.g., 21%) |
| Treatment group N at post-test | No specification (e.g., 144) |
| Pre to post effect on treatment group | 0 = Negative  1 = Positive  2 = Null |
| Which group has better outcomes? | 0 = Treatment  1 = Control  2 = Neither |
| Control group mean/SD at pretest | No specification (e.g., 4.7 (1.2)) |
| Control group % at pretest | No specification (e.g., 21%) |
| Control group N at pretest | No specification (e.g., 144) |
| Control group mean/SD at post-test | No specification (e.g., 4.7 (1.2)) |
| Control group % at post-test | No specification (e.g., 21%) |
| Control group N at post-test | No specification (e.g., 144) |
| For Odds ratio calculation (if applicable) | |
| *Variable* | *Coding details* |
| Treatment group yes | No specification (e.g., 64) |
| Treatment group no | No specification (e.g., 36) |
| Control group yes | No specification (e.g., 64) |
| Control group no | No specification (e.g., 36) |

**Appendix B. CASP Qualitative Study Assessment**

All qualitative studies included in the review will be individually assessed on the CASP (2018) qualitative study checklist, below. Additional appraisal questions may be added to this checklist, at a later date.

Studies will be rated on each question as one of the following: (1) yes, (2) can’t tell, or (3) no. Ratings decisions will be made using additional guidelines, found in the CASP (2018) checklist document.

SECTION A: Are the results valid?

1. Was there a clear statement of the aims of the research?
2. Is a qualitative methodology appropriate?
3. Was the research design appropriate to address the aims of the research?
4. Was the recruitment strategy appropriate to the aims of the research?
5. Was the data collected in a way that addressed the research issue?
6. Has the relationship between researcher and participants been adequately considered?

SECTION B: What are the results?

1. Have ethical issues been taken into consideration?
2. Was the data analysis sufficiently rigorous?
3. Is there a clear statement of findings?

SECTION C: Will the results help locally?

1. How valuable is the research?
